# Spatial Environmental Modeling of Autoantibody Outcomes among an African American Population

**DOI:** 10.3390/ijerph110302764

**Published:** 2014-03-07

**Authors:** Rachel Carroll, Andrew B. Lawson, Delia Voronca, Chawarat Rotejanaprasert, John E. Vena, Claire Marjorie Aelion, Diane L. Kamen

**Affiliations:** 1 Department of Public Health Sciences, Medical University of South Carolina, 67 President St, Charleston, SC 29425, USA; E-Mails: voronca@musc.edu (D.V.); rotejana@musc.edu (C.R.); jvena@uga.edu (J.E.V.); 2 School of Public Health and Health Sciences, University of Massachusetts Amherst, Amherst, MA 01003, USA; E-Mail: maelion@schoolph.umass.edu; 3 Department of Medicine, Division of Rheumatology and Immunology, Medical University of South Carolina, 67 President St, Charleston, SC 29425, USA; E-Mail: kamend@musc.edu

**Keywords:** lupus, autoimmunity, African Americans, environmental metals, soil, groundwater, spatial

## Abstract

In this study of autoimmunity among a population of Gullah African Americans in South Carolina, the links between environmental exposures and autoimmunity (presence of antinuclear antibodies (ANA)) have been assessed. The study population included patients with systemic lupus erythematosus (n = 10), their first degree relatives (n = 61), and unrelated controls (n = 9) where 47.5% (n = 38) were ANA positive. This paper presents the methodology used to model ANA status as a function of individual environmental influences, both self-reported and measured, while controlling for known autoimmunity risk factors. We have examined variable dimension reduction and selection methods in our approach. Following the dimension reduction and selection methods, we fit logistic spatial Bayesian models to explore the relationship between our outcome of interest and environmental exposures adjusting for personal variables. Our analysis also includes a validation “strip” where we have interpolated information from a specific geographic area for a subset of the study population that lives in that vicinity. Our results demonstrate that residential proximity to exposure site is important in this form of analysis. The use of a validation strip network demonstrated that even with small sample numbers some significant exposure-outcome relationships can be detected.

## 1. Introduction

Systemic lupus erythematosus (SLE) is a chronic autoimmune disease that, for unknown reasons, causes the immune system to attack the body’s own tissues and organs including joints, kidneys, heart, lungs, brain, blood, and skin. SLE is considered a prototypical autoimmune disease, characterized by multiple autoantibodies directed at self-antigens. Nearly 100% of patients with SLE will have antinuclear antibodies (ANA) present on serologic testing, making this a highly sensitive, albeit non-specific, screening test for SLE. Despite serious and potentially life-threatening effects, SLE is under-recognized and often goes undiagnosed for several months to years. SLE disproportionately affects young African American women [1,2]. Up to 1.5 million American are afflicted by some form of lupus, and more than five million people are known to be affected worldwide.

Environmental factors are known to influence the onset of autoimmune disorders, including SLE, among genetically susceptible individuals, however our understanding of the details of those environmental factors is limited [3]. Although first degree relatives (FDRs) of patients with SLE overall have a higher prevalence of autoantibodies and a higher risk of SLE and other autoimmune diseases [4,5], some develop SLE-specific autoantibodies but never develop clinical disease [6], implying that there are protective factors as well as additional environmental triggers that may increase the lag-time between autoimmunity and development of disease. The multifactorial nature of the genetic risk of SLE and the low disease penetrance emphasize the potential influence and complexity of environmental factors and gene-environment interactions on the etiology of SLE [7].

The SLE in Gullah Health (SLEIGH) study is a longitudinal cohort of Gullah African Americans started in 2003 to investigate potential genetic and environmental factors in the development of autoimmunity [5]. The SLEIGH study is conducted in cooperation with and approval from the Sea Island Families Project Citizen Advisory Committee [8]. The African American Gullah population is estimated to be between 100,000 and 300,000 and largely resides in the Sea Islands of South Carolina and Georgia. It is a unique community for defining environmental factors for autoimmune diseases due to its low non-African genetic admixture, environmental-geographic homogeneity within the Sea Island region, and high prevalence of ANA positivity and families with multiple incidence of SLE [5].

SLEIGH study participants were recruited to take part in a detailed assessment of lifetime residential history and estimated environmental exposures. Additionally, environmental contaminant data from soil and groundwater measurements taken from areas of South Carolina corresponding with Sea Island residential locations were obtained. Taking advantage of the data on residential histories available from the subset of SLEIGH participants, we utilized sophisticated modelling techniques to explore potential environmental factors on the development of ANA positivity among Gullah African Americans, who are known to be genetically at-risk for development of SLE. ANA is present years prior to the onset of SLE [9], thus ANA status is an ideal outcome of interest for this study.

This paper presents the methodology used to model ANA status as a function of individual environmental influences, both self-reported and measured, while controlling for known autoimmunity risk factors such as age and gender. Below we describe the data set and sampling strategy used, the modeling development procedures using the first, longest, and last residential address, and we present the results of our analysis and conclusions. 

## 2. Data Sources

### 2.1. Study Population and Exposure Questionnaires

Gullah African Americans participating in the SLEIGH study were invited between April 2010 and July 2013 to participate in an additional one-time in-person study visit where detailed lifetime exposure assessments were performed. Eighty SLEIGH study participants (61 FDRs, 10 SLE patients, and 9 unrelated controls) completed the exposure assessment visit. In this sample 47.5% (n = 38) of subjects were ANA positive A greater recruitment effort was focused on FDRs, due to their known increased risk for developing SLE over that of the general population and therefore the relevance of ANA positivity as a potential biomarker predictive of future progression from silent autoimmunity to clinically significant autoimmune disease. The SLEIGH study and all the methodology described here were conducted with the approval of the MUSC Institutional Review Board for Human Subjects Research and the Sea Island Families Project Citizen Advisory Committee [5,8]. The residential addresses of these participants vary during the study period, and so to simplify the analysis of residency, we have examined three key addresses which could impact exposure windows: First recorded address (birth), longest address (address for which the participant resided longest), and last address (the most recent address currently reported). These addresses correspond to early exposure, extended or cumulative exposure and recent exposure, respectively. Additional personal participant variables have been included in the analysis based on in-person study visit assessments and questionnaire responses. The study questionnaires included a detailed residential and occupational history, questions about diet (including local seafood consumption), ascertainment of lifestyle factors (including well water use, smoking status, pesticide use) and health questions (including medication history). The survey was developed based on the experience of two prior studies of environmental exposures and SLE, the Buffalo Lupus Study and the Carolina Lupus Study, and validated for use within the Gullah African American community [7,10,11,12,13]. These variables are listed in Table 2. 

### 2.2. Environmental Contaminant Databases

The ground water and soil chemical survey data were measured in 2005 and made available by the United States Geological Survey (USGS) [14]. The strip data used for validation were made available by Professor Claire Marjorie Aelion, of the University of Massachusetts Amherst. These data consist of metal concentrations measured in soil samples taken from a relatively dense network of sites which were originally established for the analysis of soil metals and childhood neurological outcomes withither study (NIEHS: ES012895-04A2). The strip was sampled in 2011. The accuracy of Kriged estimates in the original study is discussed in [15]. With respect to participants in the strip, 8 people out of 14 people were diagnosed with the positive ANA status at the first address, 9 out of 15 and 6 out of 10 people were ANA positive at the longest and last addresses. Both the USGS and strip data made use of heavy metals, pesticides, and organochlorines in the ground water and/or soil.

## 3. Data Quality

While exposure assessment is ideally performed prospectively and at a local or individual level, it is not always possible to achieve this goal due to feasibility and cost and especially for rare outcomes such as autoimmunity. Instead, it is often necessary to use a retrospective study design and without direct measurement of intake, to use exposure surrogates. In our study, we have the location of different residential addresses for members of the cohort and control populations but we do not have precisely contemporaneous soil or groundwater metal measurements. In addition we do not have precise measurements of exposure to chemical measurements at residential locations. Instead, we have self-report addresses for different periods in the lifetime of the subjects, and measures of soil and groundwater chemicals made at a network of locations and at one time (2005). This 2005 measurement is considered an average over time since the measures could be varying either before or after. As addresses range across the measurement year we must assume a “window of risk” around that year. The network of sites measured does not closely correspond with address locations of participants. This misalignment of locations was allowed for by adopting a functional relationship between residential location and chemical measurement site. We have formed a set of distance-modified soil and groundwater chemical exposure measures. These are detailed more fully in the next section. 

## 4. Modeling Approaches

Each of the participants in the study has a residential address at a given time. The number of different addresses varies across participants and so to simplify analysis we have examined three main addresses for each participant: birth address (first), the address where they resided the longest (longest), and current address (last). In our analyses we have used these addresses so that in all instances. Our analyses have been carried out for each of these addresses separately. Our outcome of interest was *ANA status*, a binary outcome denoting whether a participant is ANA positive (ANA titer > 1/40) or not. 

For discrete ANA status we assume a logistic spatial model as follows:

*y_i_*~*Bern*(*p_i_*)

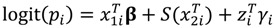

where the fixed design matrix includes a range of parameters both personal and environmental with *i*th element 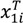 corresponding to the *i*th individual. The prior distributions for regression parameters, *β*, are assumed to be zero mean Gaussian such that *β*~*N*(0,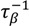) with a gamma prior distribution for the precisions, *τ_β_*~*Ga*(1,5*e* − 05) for each **β** independently, except when variable selection is employed. Using first order random walks we also included smoothing of a subset of predictors 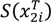. For the random component, we assume that *γ* represents an individual level random effect, and that 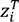 is a binary indicator vector of length *m*, the number of individuals. This is essentially a random intercept per individual such that the prior distribution is *γ_i_*~*N*(0,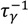) with a non-informative gamma prior distribution for the precision, *τ_γ_*~*Ga*(1,5*e* − 05).

Within the design matrix issues exist regarding the number of parameters with the limited sample size. Two approaches were implemented to resolve this issue: variable dimension reduction and variable selection.

First, we considered a dimension reduction strategy whereby we focused on the set of chemical measures and their corresponding underlying components. The purpose of this was to derive a smaller set of components which could be used as regressors within any model. We conducted a Principal Component Analysis (PCA) [16] of the subset of chemical measures, both singly for soil chemicals and groundwater (GW), and also jointly with the soil and GW subset combined. This aided in reducing the number of parameters that reside within **β** by creating a score based on the correlations among the environmental metal measures to use in lieu of the set of chemical measures. We used the correlation matrix of the chemicals rather than the covariance in this PCA to allow for different variability in the measures. Often we found that only one or at most two components explained >80% of the variation, 80% is the significance criterion [17]. In the candidate models used in all subsequent analyses we have considered either PCA scores for chemicals or the set of chemicals related to the individual through distance in a given model.

Second, performing Bayesian variable selection with both optional linear and non-linear link functions in generalized additive mixed models [16] also leads to a reduction in the number of variables based on the significance of their relationship to the outcome of interest. This procedure employs a Normal-mixture of inverse Gammas (NMIG) prior to determining which covariates as factors, penalized B-splines, or linear effects should be used in the model without having to calculate marginal likelihoods. This NMIG results in a spike-slab like prior on the coefficients **β**, by supplying a bimodal prior on the variance, *τ*^2^*η*, of those coefficients. The spike and slab posterior weights,*η*, can then be interpreted to determine the inclusion or exclusion of the parameter. This application is specified as follows:

*β*|*η*, *τ*^2^~*N*(0, *τ*^2^*η*)

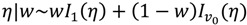

*τ*^2^~*Γ*^−1^(*a_τ_*, *b_τ_*); *w*~*Beta*(*a_w_*, *b_w_*)

where I*_x_*(*η*) represents an indicator function that is 1 in x and 0 elsewhere and *v*_0_ is a small, positive constant such that the indicator *η* is 1 with probability *w* and 0 with probability 1 − *w*. Thus if *η* = *v*_0_, the variance is very small creating the spike component of the prior. *Γ*^−1^ denotes an inverse Gamma prior for *τ*^2^. We have employed the R package spikeSlabGAM [17] (SSG) for this purpose. Additionally, SSG has the ability of including random effects [17]. Once the inclusion probability for a variable is derived an inclusion threshold for 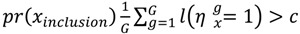 from the converged sample of G parameter values is assumed. Usually a minimum value for inclusion is *c* = 0.5 [18].

## 5. Validation Study

**Figure 1:**
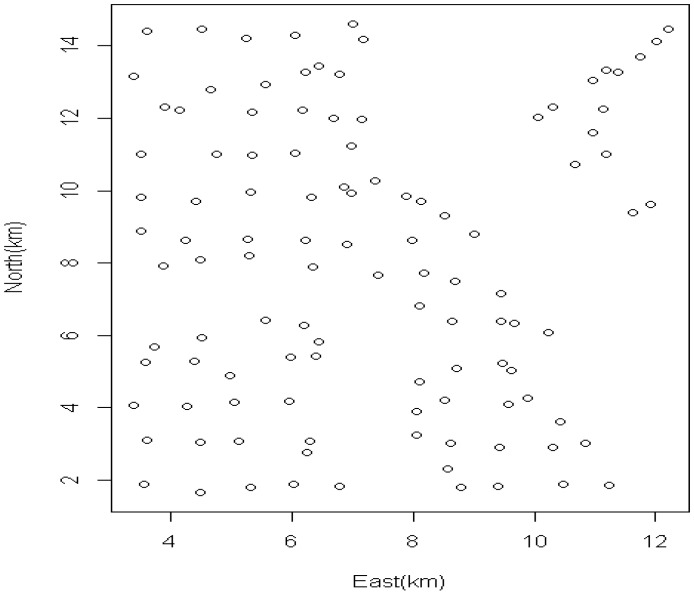
Spatial distribution of sampling sites in the validation strip area.

To provide a validation for the distance metric exposure models we decided to examine a dataset which involved exposure assessment via spatial interpolation. For the validation study we have used a sampling strip which consists of a network of 110 sites where a range of soil metals has been measured. The strip was sampled in 2011. Figure 1 displays the map of the sampling sites. The sampling strip provides more detailed spatial coverage of an area close to many of the addresses of study participants. Because the strip has a relatively dense network of sites we can employ Bayesian Kriging [19] to interpolate chemical measures to the sites of participant addresses. A small number of participants lived on or near the strip. We also include those who were located within 1 km of the outer strip boundary as the interpolation error was found to remain small up to that range. Descriptive statistics of the subjects that fit these criteria are included in Table 1, and these statistics demonstrate that the validation sample well represented the full data set.

**Table 1:** Descriptive statistics associated with the validation study sample compared to the full data set.

Sample	% ANA Positive	% Male	Median Age
First address (n = 14)	57%	14.3%	54
Longest address (n = 15)	60%	<1%	54
Last address (n = 10)	60%	10%	57.5
Full Data Set	47.5%	15%	54

In our modeling of the participant outcomes for the strip we employ a 2 stage approach. We first perform an interpolation of chemicals to the addresses. Then we include a random additive measurement error component in our health model (*ω_ij_*) so that

*y_ij_*~*Bern*(*p_ij_*)

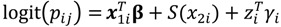

where 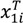 is a fixed design matrix, 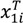
**β** is a linear predictor, and 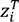
*γ_i_* is a random effect assumed to have a zero-mean Gaussian prior distribution alike our previous model definitions. The definition of the predictor function is innovative as we assume that *S*(*x*_2_*_i_*) can have a range of forms. In this study we limit the link functions to random walk smoothing akin to B-splines [20], to allow for flexible functional dependence on the measured chemicals and personal variables. 

## 6. Results

**Figure 2:**
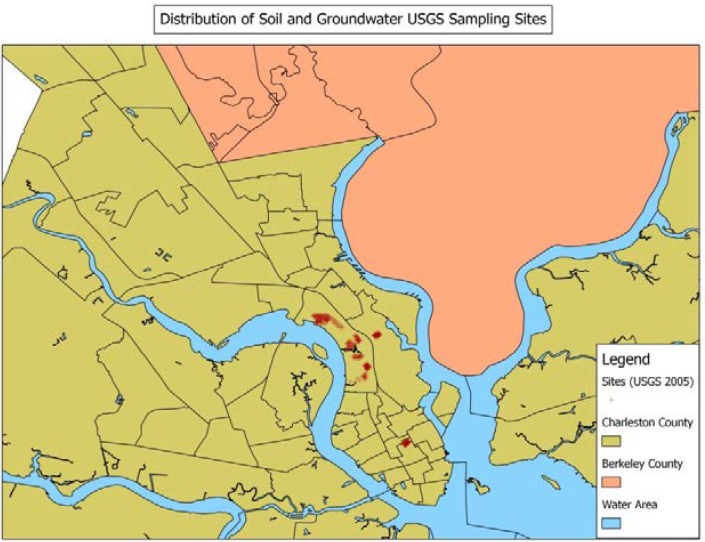
Spatial distribution of soil and groundwater sampling sites.

**Figure 3:**
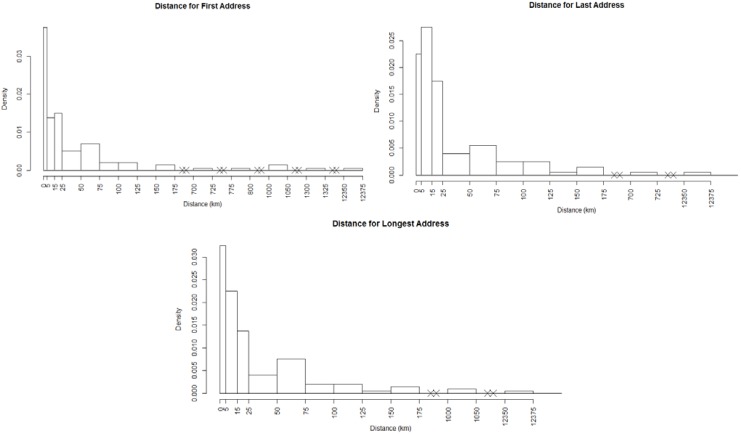
Histograms of address distances to soil mercury sampling sites for first, last and longest addresses.

Figure 2 displays the main sampling sites for soil and groundwater in the study. For confidentiality reasons we cannot display the residential addresses of the participants. Figure 3 displays the histograms of the distance of participants from the mercury measures at soil sampling sites. Similar distributions are realized for other soil and groundwater chemicals measured at their respective sites also but are not shown. Predominantly distances within 15 km are displayed for all scenarios. Figure 1 displays the distribution of the 110 sample locations. The design of the sites in that study is detailed elsewhere [21,22].

**Figure 4:**
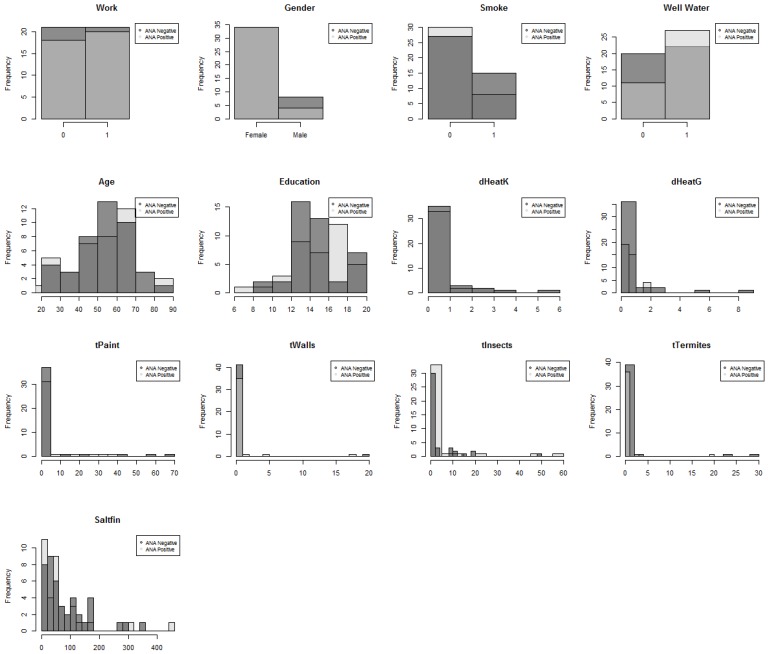
Distribution of personal variables with respect to ANA status.

Table 2 displays the variables, both chemical and personal, that were used in our model building process. The personal variables include demographics (age, gender, education level), lifestyle and behavioral survey responses (smoking, working status, well water consumption, fish consumption), and living conditions (termite treatment, replacement of walls, painting of house, kerosene or gasoline heating). Figure 4 displays the distribution for the personal variables listed in Table 2 with respect to ANA status. 

**Table 2:** Individual level and chemical variables applied in the study with associated descriptions.

Variable	Definition
tTermites	Times the individual’s home was treated for termites
tInsects	Times the individual’s home was treated for insects
tWalls	Times the individual tore down walls
tPaint	Times the individual worked with paint
education	Number of years of education
CurAge	Current age of the individual
dHeatK	Exposure to a kerosene heater
dHeatG	Exposure to a gasoline heater
Work	Individual works more than 10 hours a week, binary
Smoke	Individual a smoker, binary
gendernum	Individual gender, binary
Saltfin	Individual fish consumption per year
well_water	Individual uses well water, binary
Mercury	Soil (µg/kg) and groundwater (µg/L) mercury sample measures
Arsenic	Soil (µg/kg) and groundwater (µg/L) arsenic sample measures
Lead	Soil (µg/kg) and groundwater (µg/L) lead sample measures
triCE	Soil (µg/kg) and groundwater (ug/L) 1,1,1-Trichloroethane sample measures
tetraCE	Soil (µg/kg) 1,1,2,2-Tetrachloroethane sample measures
triCE112	Soil (µg/kg) 1,1,2-Trichloroethane sample measures
Phth	Soil (µg/kg) Chloronaphthalene sample measures
Acetone	Soil (ug/kg) and groundwater (µg/L) acetone sample measures
Dintolu	Soil (µg/kg) and groundwater (µg/L) 2,4-Dinitrotoluene sample measures
Dintolu26	Soil (µg/kg) 2,6-Dinitrotoluene sample measures
Endo2	Soil (µg/kg) and groundwater (µg/L) Endosulfan 2sample measures
Endo1	Soil (µg/kg) and groundwater (µg/L) Endosulfan 1sample measures
Toluene	Soil (µg/kg) and groundwater (µg/L) toluene sample measures
DDT	Soil (µg/kg) and groundwater (µg/L) DDT sample measures
Atrazine	Soil (µg/kg) and groundwater (µg/L) atrazine sample measures
Tribenz	Soil (µg/kg) and 1,2,4-Trichlorobenzene sample measures
Dibenz	Soil (µg/kg) and 1,2-Dichlorobenzene sample measures
Benz	Groundwater (µg/L) robenzene sample measures
Biphen	Groundwater (µg/L) 1,1'-Biphenyl sample measures
Endosulf	Groundwater (µg/L) Endosulfan sulfate sample measures
Dinphth	Groundwater (µg/L) Di-n-butylphthalate sample measures
Clphth	Groundwater (µg/L) Chloronaphthalene sample measures
As	Arsenic soil (mg/kg) sample measures from the strip validation study data
Ba	Barium soil (mg/kg) sample measures from the strip validation study data

In the initial analysis we performed a PCA of distance weighted soil, groundwater (GW), and combination of soil + groundwater chemicals. The distance weighting was of the form 
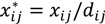
 and 
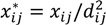
, where *d_ij_* is the distance from the residential address of the participant to the sample site of the chemical calculated using the spherical law of cosines. Note that this distance can vary depending on whether the first, longest or last address is used. This transformation represents an inverse linear and inverse quadratic weighting of the variables. Figure 3 displays the histograms of the distance distributions for each address class (first, longest, and last). All chemicals were transformed in this way prior to all subsequent analysis. 

Table 3 displays the PCA loadings as well as the direction of the loadings for each of the significant components broken down by first, longest and last addresses as well as soil only, ground water only, and the joint of soil and ground water. The direction of the loadings can aid in interpreting the PCA component if it is significant in the model. For example, if the chemical loads positively (+) and the parameter estimate associated with that component is also positive, then the chemical has a positive relationship with ANA status. In most instances only one component was found to explain over 80% of the variation (in soil and GW). In the joint analysis two components were often found. In all instances for the majority of the analyses, the same chemicals were selected across the analyses. Once the components were derived these were used in subsequent logistic regression modeling.

The next stage in the analysis was to assess the importance of a variety of distance weighted chemicals, chemical Principal Component scores (PCs) and personal variables in the explanation of ANA status. Initially, we examined single predictor models (chemicals, PCs and personal variables), but decided for efficiency to employ variable selection algorithms to find the most important contributions to models. To this end, we employed Bayesian variable selection using spike and slab prior distributions (Stochastic Search Variable Selection) [23]. In our full models we included all personal variables with either PCs or the set of individual chemicals. These models were fitted for each of the address variants (first, longest, and last) separately. 

Table 4 displays the variable selection results depicting the variables that were found to have a c > 0.25; the variables that are considered “important” satisfy the c = 0.5 inclusion criterion [18]. Many of the variable selection runs resulted in choosing either the null or random intercept-only models. None of the personal variable, chemical, or PCs covariates met the inclusion criterion. Furthermore, the variables that do appear in the table have quite large standard deviations meaning that they are not even well estimated to be above 0.25. Notice also that typically, when a chemical appears in the table, it appears in the longest address section. This suggests that exposure time to the chemical could be important. Based on these results, after the implementation of PCA and variable selection, no covariates met the inclusion criterion. Thus, the analysis of soil and GW did not present any covariates to be included in a predictive model for measuring associations with ANA status.

Finally, Table 5 displays the results of the Kriging validation. This displays the variables that met the inclusion criterion of “important” where c = 0.5 as seen previously [18]. Notice that many of these variables also have fairly large standard deviation values, but are still better estimated than the models presented in Table 4. The table shows their mean inclusion probability and standard deviation as well as the associated parameter estimates and 95% credible interval. The analysis of ANA status in relation to Kriged soil chemicals demonstrates that the soil measures used in this part of the analysis are better at capturing the true association of the selected variables to ANA status. Here, lead and chromium related positively to ANA status while copper related negatively. We did not find any well estimated personal variables in the strip analysis.

**Table 3:** PCA loadings and directions (+/−) for the first, longest, and last addresses in that order broken down by soil only (S), ground water only (W), and the joint of soil and ground water (S+G). See Table 2 for description of the variable names.

	Distance		Distance Squared	
	No. of Comps	Loading	No. of Comps	Loading
**First Address**				
S	1	1: mercury(−), lead(−), dintolu(−), dintolu26(−), atrazine(−), tribenz(−), dibenz(−)	1	1: mercury(−), dintolu(−), dintolu26(−), atrazine(−), tribenz(−), dibenz(−)
W	1	1: Arsenic(−), Lead(−)	1	1: Arsenic(−), Lead(−)
S+W	2	1: all negative except leadW didn’t load at all 2: mercury S(−), arsenicS(−), triCES(−), tetraCE(−), triCE112(−), acetone(−), endo2S(−), endo1S(−), tolueneS(−), DDTS(−), mercuryW(+), arsenicW(+), leadW(+), endo2W(+), endo1W(+), DDTW(+), endosulfW(+)	2	1: all negative except leadW didn’t load at all 2: mercury S(−), arsenicS(−), leadS(−), tetraCES(−), triCES(-), triCE112S(−), acetoneS(−), endo2S(−), endo1S(−), tolueneS(−), DDTS(−), mercuryW(+), arsenicW(+), leadW(+), endo2W(+), endo1W(+), DDTW(+), endosulfW(+)
**Longest Address**				
S	2	1: mercury(−), dintolu(−), atrazine(−), tribenz(−), dibenz(−) 2: mercury(−), lead(−), dintolu(+), dintolu26(−), atrazine(−), tribenz(−), dibenz(−)	2	1: mercury(−), lead(−), dintolu(−), dintolu26(−), atrazine(−), tribenz−), dibenz(−) 2: lead(−), dintolu(−), dintolu26(−), atrazine(−), tribenz(−), dinbenz(−)
W	1	1: Arsenic(−), Lead(−)	1	1: Arsenic(−), Lead(−)
S+W	2	1: all negative except leadW didn’t load at all 2: mercury S(−), tetraCES(+), triCES(+), dintoluS(−), endo2S(+), endo1S(+), tolueneS(−), DDTS(+), mercuryW(−), arsenicW(−), leadW(−), acetoneW(+), endo2W(−), endo1W(−), DDTW(−), endosulfW(−)	1	1: all negative except leadW didn’t load at all
**Last Address**				
S	2	1: mercury(−), lead(−), dintolu(−), atrazine(−), tribenz(−), dibenz(−) 2: mercury(−), lead(−), dintolu(+), atrazine(−), tribenz(−), dibenz(−)	2	1: mercury(−), lead(−),dintolu(−), atrazine(−), tribenz(−), dibenz(−) 2: mercury(−), lead(−),dintolu(+), atrazine(−), tribenz(−), dibenz(−)
W	1	1: Arsenic(−), Lead(−)	1	1: Arsenic(−), Lead(−)
S+W	2	1: all negative 2: mercuryS(−), arsenicS(−), leadS(−), dintoluS(+), tolueneS(+),artrazineS(−),dibenzS(−), mercuryW(+), arsenicW(+), leadW(+), acetoneW(−), endo2W(+), endo1W(+), tolueneW(−), DDTW(+),endosulfW(+)	2	1: all loaded negative 2: mercury S(−), arsenicS(−), leadS(−), triCES(−), tetraCES(−), acetoneS(+), dintoluS(+),endo2S(+), endo1S(+), tolueneS(+), DDTS(+), mercuryW(+), arsenicW(+), leadW(+), acetoneW(−), endo2W(+), endo1W(+), tolueneW(−), DDTW(+), endosulfW(+)

**Table 4:** The posterior mean and standard deviation of the inclusion probability for variable selection algorithms applied to first, longest, and last addresses presented in that order. Rnd (id2) here indicates the random intercept component of the model.

	Distance		Distance Squared	
	Parameter	Inclusion Probability Mean (sd)	Parameter	Inclusion Probability Mean (sd)
**First Address**				
PCA				
Soil	Rnd(id2)	0.326 (0.469)	Rnd(id2)	0.334 (0.472)
GW	Rnd(id2)	1.000 (0.000)	Educ	0.337 (0.473)
	---	---	Rnd(id2)	0.668 (0.471)
Joint	Rnd(id2)	0.334 (0.472)	Rnd(id2)	0.667 (0.471)
Chemical				
Soil		NULL	Rnd(id2)	0.667 (0.471)
GW	Rnd(id2)	0.334 (0.472)	Rnd(id2)	0.334 (0.472)
Joint	Rnd(id2)	0.667 (0.471)	Rnd(id2)	0.667 (0471)
**Longest Address**				
PCA				
Soil	Rnd(id2)	0.667 (0.471)	Rnd(id2)	0.667 (0.471)
GW	Rnd(id2)	0.334 (0.472)	Rnd(id2)	0.334 (0.472)
Joint	Rnd(id2)	0.667 (0.471)	Rnd(id2)	1.000 (0.000)
Chemical				
Soil	Rnd(id2)	1.000 (0.00)	tetraCE	0.346 (0.476)
	---	---	Educ	0.334 (0.472)
	---	---	Rnd(id2)	0.334 (0.472)
GW	Biphen	0.294 (0.456)	Rnd(id2)	0.667 (0.471)
	Rnd(id2)	0.334 (0.472)	---	---
Joint	AtrazineW	0.334 (0,472)	tribenzS	0.334 (0.472)
	Rnd(id2)	0.334 (0,472)	Educ	0.334 (0.472)
	---	---	Rnd(id2)	0.334 (0.472)
**Last Address**				
PCA				
Soil	Rnd(id2)	0.334 (0.472)	Rnd(id2)	0.667 (0.471)
GW	Rnd(id2)	1.000 (0.000)	Rnd(id2)	0.334 (0.472)
Joint	Rnd(id2)	0.667 (0.471)	Rnd(id2)	0.667 (0.472)
Chemical				
Soil	Atrazine	0.334 (0.472)	Rnd(id2)	0.667 (0.471)
	Rnd(id2)	0.334 (0.472)	---	---
GW	Rnd(id2)	0.334 (0.472)	Rnd(id2)	1.000 (0.000)
Joint	Rnd(id2)	0.667 (0.471)	NULL	NULL

**Table 5:** Inclusion probability posterior mean and standard deviation as well as mean parameter estimate and 95% credible interval from Kriging broken down by first, longest, and last address from the validation strip.

	Birth Address		Longest Address		Last Address	
Parameter	Inclusion probability Mean (sd)	Parameter Estimate Mean (95% CI)	Inclusion probability Mean (sd)	Parameter Estimate Mean (95% CI)	Inclusion probability Mean (sd)	Parameter Estimate Mean (95% CI)
Age	---	---	---	---	0.5585 (0.4966)	−3.049 (−10.56, 0.203)
dheatG	---	---	---	---	0.5540 (0.4971)	−3.575 (−14.94, 3.881)
tPaint	---	---	---	---	0.5796 (0.4936)	−2.413 (−15.5, 6.809)
tTermites	0.5664 (0.4956)	−2.507 (−15.31, 8.914)	0.7076 (0.4549)	−4.307 (−18.04, 8.093)	---	---
Cr	0.6298 (0.4829)	4.739 (0.005, 15.81) *	---	---	---	---
Cu	0.6426 (0.4792)	−2.377 (−8.386, −241) *	---	---	---	---
As	---	---	0.6166 (0.4862)	0.862 (−13.76, 14.56)	---	---
Mn	---	---	0.7096 (0.4540)	0.116 (−650, 1.054)	---	---
Pb	---	---	---	---	0.6098 (0.4878)	2.844 (0.320, 9.006) *

Note: * Indicates a well estimated variable.

## 7. Discussion and Conclusions

Many lines of evidence point to environmental factors playing a significant role in triggering autoimmunity in individuals with a genetic predisposition. Although the role of specific environmental factors and the mechanisms by which they act remain poorly understood, identification of influential environmental exposures, including soil and groundwater contaminants, will help inform future studies and exposure evaluation methods. 

There are several limitations to the complex methodology presented here including distance estimations and the large distances between the sample sites. If these data were more finely collected, we may be able to get a better measure of the associations to ANA status by employing Kriging methods presented in our validation study. Furthermore, if we could get chemical data measures from the actual participant addresses, we might have even greater confidence in establishing associations between exposure and outcome. Another issue with all studies based on survey data is bias from many different sources though random effect methods were employed to reduce the influence of these biases. The greatest limitation for this study is the small sample size. If we were able to apply more subjects to the study methodology, we may have been able to find even more association with ANA status. This limited sample size and the fact that our subjects are all Gullah African American also hinders our generalizability to other populations that might have more of a genetic admixture present.

The misalignment of locations could have been allowed for via interpolation of chemicals to residential addresses [19,24] rather than allowing a functional relationship between residential location and chemical measurement site. The first approach is appropriate when a reasonably fine network of sites covers the study area. We do not have a large number of sites, and they are irregularly distributed. Thus, we adopted a distance-based approach to exposure modeling.

Although sensitive and specific biomarkers of exposure and disease continue to be discovered and utilized, the majority of environmental risk studies to date rely on questionnaires to ascertain exposure and/or outcomes of interest. Advantages of utilizing data from the SLEIGH study include the use of questionnaires and other assessments which were designed and validated to formally assess environmental exposures of interest and autoimmune disease outcomes. However, these current methods of estimating environmental exposure are limited by an excessively long lag-time between time of exposure of interest and time of assessment, particularly problematic in light a long pre-clinical phase in SLE and in today’s rapidly changing environment.

In this study we have examined a range of possible methods that can be applied to environmental data that have variable temporal and spatial resolutions. These approaches are quite innovative and could be applied in a variety of settings using longitudinal data with spatial characteristics. The methodology presented here demonstrates how meticulously collected exposure data can be used in conjunction with even a relatively small well-characterized population to discover potential environmental influences on the development of ANA positivity among genetically at-risk individuals. Comparing the final model to the validation study shows how important meticulous exposure data collection can be. With the more meticulously collected exposure data we were able to find chemicals associated with ANA status. 

Our findings emphasize the importance of efforts to continue refining these sophisticated modeling techniques and to include larger numbers of well-characterized individuals with both detailed exposure and outcome data available. These efforts could ultimately lead to novel prediction tools to identify individuals most likely to develop SLE-related autoimmunity and could inform efforts to prevent progression to autoimmune disease.
